# Electroacupuncture alleviates cognitive impairment in mice with vascular dementia by regulating the cholinergic vasodilation system

**DOI:** 10.3389/fnagi.2026.1686345

**Published:** 2026-02-13

**Authors:** Yuanjie Gao, Jianxin Zhao, Jianpeng Chen, Rongming Qi, Yuxuan Yuan, Bohong Liu, Xiaohan Yu, Yaru Liu

**Affiliations:** 1 Shunyi Hospital, Beijing Traditional Chinese Medicine Hospital, Beijing, China; 2 School of Acupuncture-Moxibustion and Tuina, Beijing University of Chinese Medicine, Beijing, China; 3 Department of Acupuncture and Moxibustion, Beijing University of Chinese Medicine Third Affiliated Hospital, Beijing, China; 4 Beijing University of Chinese Medicine, Beijing, China; 5 School of Traditional Chinese Medicine, Beijing University of Chinese Medicine, Beijing, China

**Keywords:** acetylcholine, cholinergic vasodilation system, electroacupuncture, m-1AChR, neurovascular coupling, vascular dementia

## Abstract

**Aims:**

Electroacupuncture (EA) is a potential and reliable treatment for vascular dementia (VaD). This study aims to explore the mechanism of EA in the treatment of VaD based on neurovascular coupling.

**Methods:**

The VaD model was established by bilateral common carotid artery occlusion (BCCAO). Mice received EA at Baihui (GV20), Geshu (BL17), and Shenshu (BL23) acupoints for 7 days. Then, behavioral tests, leptomeningeal microvascular observation, immunofluorescence of basal forebrain neurons, and molecular analyses of the ACh/NO pathway were conducted.

**Results:**

EA stimulation alleviated cognitive impairment in VaD mice, dilated leptomeningeal arterioles, enhanced neuronal activity and acetylcholine expression in the basal forebrain, and increased the levels of m-1AChR, α-7-nAChR, eNOS, and NO in the frontal cortex. The m-1AChR antagonist (THP), but not the α-7-nAChR antagonist (MLA), partially reversed these regulatory effects of EA.

**Conclusion:**

EA dilates cerebral arterioles by up-regulating the ACh/NO pathway within cholinergic vasodilation system, thereby alleviating cognitive impairment induced by VaD.

## Graphical abstract

**Figure:**
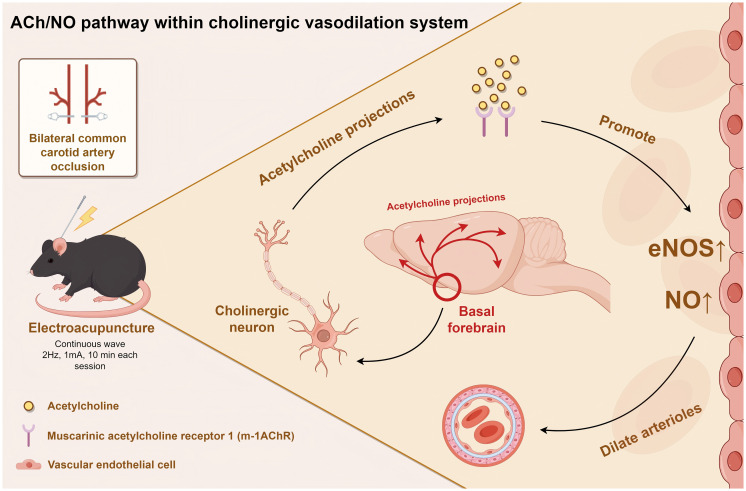
Electroacupuncture dilates cerebral arterioles by up-regulating the ACh/NO pathway within cholinergic vasodilation system, thereby alleviating cognitive impairment induced by vascular dementia. This image was made by figdraw.com. ACh, acetylcholine; eNOS, endothelial nitric oxide synthase; NO, nitric oxide.

## 1 Introduction

Vascular dementia (VaD) is a severe type of vascular cognitive impairment, which is the second most common type of dementia after Alzheimer’s disease (AD) (Wolters and Ikram, 2019). Compared to AD, VaD is associated with a shorter survival time and poorer long-term prognosis (Liang et al., 2021).

As research progresses, it is increasingly recognized that hypoperfusion caused by cerebral small vessel disease is one of the key pathways in the pathogenesis of VaD (Inoue et al., 2023). Small brain lesions alter cerebral microvascular hemodynamics, leading to aggregation of red blood cells (RBCs), white blood cells, and platelets, which form microthrombi. Subsequently, the interaction between white blood cells and vascular endothelial cells alters blood–brain barrier permeability and causes perivascular edema, worsening hypoperfusion and neuronal damage. The normal physiological functions of brain rely on the close coordination between neural activity and hemodynamics to regulate local cerebral blood flow (CBF) distribution. Microvessels respond to neuronal activity by dilating or constricting, ensuring precise local blood supply. This phenomenon is known as “neurovascular coupling (NVC)”. Cerebral small vessel disease impairs the basal forebrain cholinergic pathway, reducing vasodilatory activity and disrupting CBF regulation (Wan et al., 2019; Caruso et al., 2019). Therefore, regulating microvascular dilation and improving cerebral perfusion are essential for the prevention and treatment of VaD.

Current pharmacological treatments for VaD include cholinesterase inhibitors, excitatory amino acid receptor antagonists, nimodipine, and citicoline (Chang Wong and Chang Chui, 2022). Studies indicated that these medications provide only slight and transient benefits in mild to moderate VaD, with side effects like nausea, vomiting, irritability, and dizziness (Shi et al., 2023; Sun, 2018). An increasing number of high-quality studies have found that acupuncture can improve cognitive function and daily living abilities in VaD patients with minimal side effects (Liu et al., 2016; Shi et al., 2015; Shi et al., 2023), offering a beneficial complement to current treatments.

Electroacupuncture (EA) is a commonly used acupuncture method in traditional Chinese medicine practice, which combines needle stimulation with electrical stimulation. Using appropriate parameters can achieve the effect of sustained needle manipulation, thereby enhancing the therapeutic efficacy of acupuncture. Previous studies have found that EA can improve cognitive function through multiple pathways, including the regulation of neurotransmitters (Soligo et al., 2017), inhibition of inflammatory responses (Bu et al., 2022), resistance to neuronal apoptosis (Ma et al., 2022), facilitation of synaptic plasticity (Dai et al., 2022), and modulation of free radicals (Wang et al., 2004). Notably, EA also alleviates cognitive impairment by promoting angiogenesis (Yang et al., 2025), repairing microvascular structure (Du et al., 2023), and activating the locus coeruleus—prefrontal cortex noradrenergic circuit (Wan et al., 2025) to improve CBF.

As an important neurotransmitter in the brain, acetylcholine (ACh) participates in maintaining the basal vasomotor tone in the brain parenchyma, and it is also a crucial mediator of the NVC effect (Lecrux et al., 2017). All ACh in the neocortex is derived from cholinergic neurons in the basal forebrain. Studies have shown that stimulating basal forebrain cholinergic neurons to release ACh increased the cortical CBF (Lecrux et al., 2017). The binding of ACh to acetylcholine receptors can induce the activation of endothelial nitric oxide synthase (eNOS), leading to an increase in nitric oxide (NO) content, which in turn triggers vasodilation and improves CBF (Nizari et al., 2021). NVC emphasizes the structural connections and functional interactions between nerves and vessels. Its multi-level, multi-pathway network structure conforms to the holistic regulatory characteristics of acupuncture.

Acetylcholine receptors are divided into muscarinic acetylcholine receptors (mAChRs) and nicotinic acetylcholine receptors (nAChRs). It has been found that an mAChR blocker (atropine) can eliminate the enhancement effect of EA on cerebral perfusion in mice with cerebral ischemia (Kim et al., 2013). However, findings have been inconsistent regarding whether α-7-nicotinic acetylcholine receptor (α-7-nAChR), a specific subtype of nAChRs, mediates the dilation of cortical arterioles (Cheng et al., 2011; Kim et al., 2013; Lee et al., 2011; Nakajima et al., 2003). Therefore, we selected trihexyphenidyl hydrochloride (THP), a specific muscarinic acetylcholine receptor 1 (m-1AChR) antagonist, and methyllycaconitine citrate (MLA), a specific α-7-nAChR antagonist, as inhibitors to block the ACh/NO pathway within the cholinergic vasodilation system.

**Figure 1:**
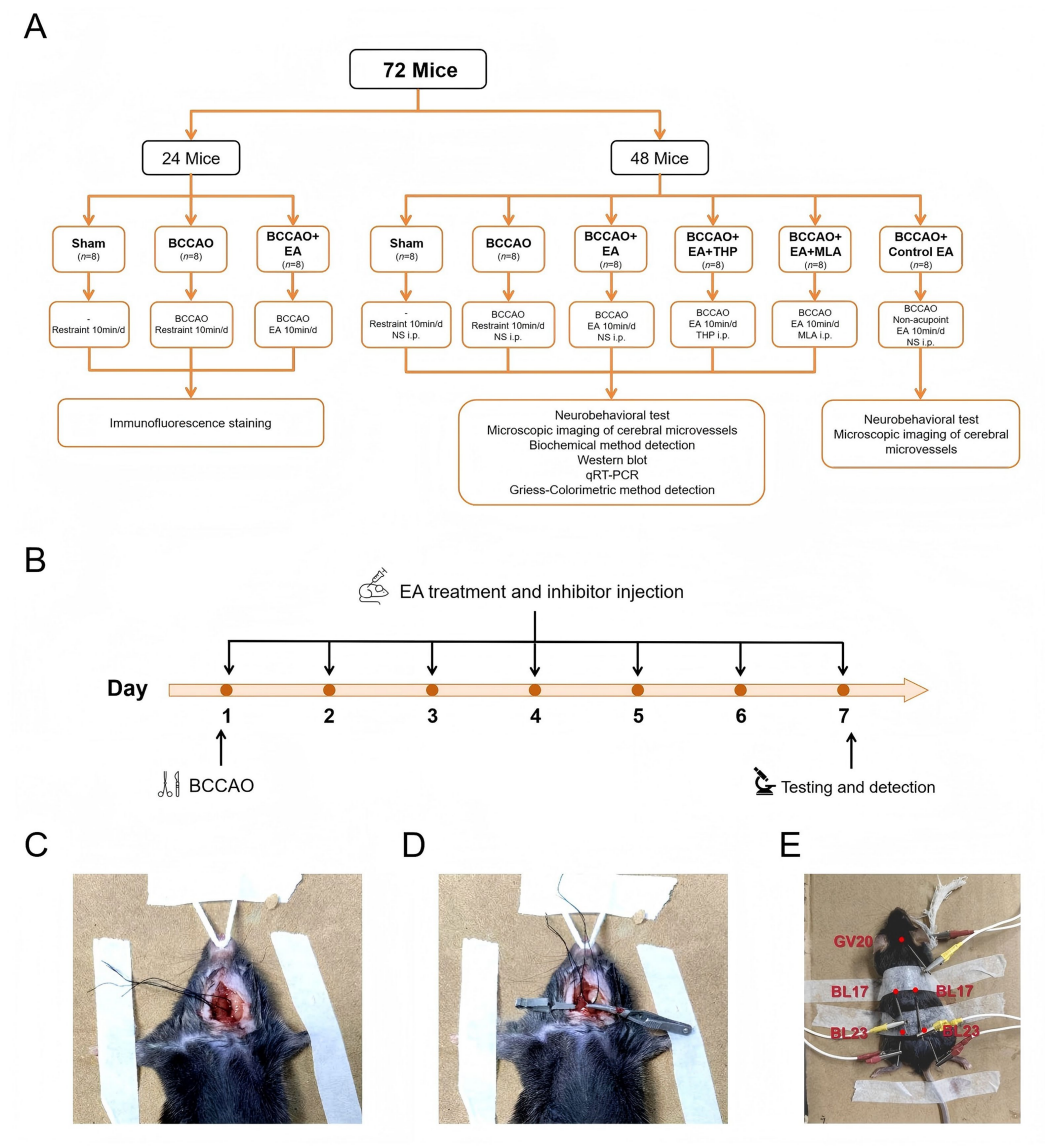
Experimental procedure. **(A)** Animal grouping and intervention protocols. **(B)** Experimental timeline. **(C)** The mouse VaD model was established using BCCAO surgery. The common carotid arteries (CCAs) and accompanying vagus nerves were carefully separated. 4-0 sutures were looped around the CCAs for later use. **(D)** The CCAs were clamped with vascular clips for 20 min to block blood flow. **(E)** The mouse was stimulated at Baihui (GV20), Geshu (BL17), and Shenshu (BL23) acupoints. GV20 is located at the intersection point of the midline of the vertex and the line connecting the apex of both ears. BL17 is located 3 mm lateral to the dorsal midline, at the lower edge of the spinous process of the 7th thoracic vertebra. BL23 is located 3 mm lateral to the dorsal midline, at the lower edge of the spinous process of the 2nd lumbar vertebra. The negative electrode of the EA instrument was connected to the GV20 needle. The positive electrode was clipped to a small piece of saline-moistened gauze wrapped around a forelimb. The ipsilateral BL17 needle (positive electrode) was connected to the BL23 needle (negative electrode). BCCAO, bilateral common carotid artery occlusion; EA, electroacupuncture; THP, trihexyphenidyl hydrochloride; MLA, methyllycaconitine citrate; NS, normal saline; i.p., intraperitoneal injection; qRT-PCR, quantitative real-time polymerase chain reaction; VaD, vascular dementia.

Our previous study found that EA at Baihui (DU20), Geshu (BL17), and Shenshu (BL23) acupoints improved the cerebrovascular morphology, decreased neuronal apoptosis, and ameliorated the learning and memory functions in VaD mice (Liu et al., 2023; Liu, 2018). In this study, we focused on NVC and hypothesized that EA alleviates VaD-induced cognitive impairment by regulating the cholinergic vasodilation system. We established the mouse VaD model using bilateral common carotid artery occlusion (BCCAO) surgery. The objectives of our study are: (1) to evaluate the therapeutic efficacy of EA for VaD, (2) to observe the effects of EA on cerebral microvessels and basal forebrain neuronal activity in VaD mice, and (3) to explore the regulatory effect of EA on the ACh/NO pathway within the cholinergic vasodilation system in VaD mice. The experimental procedure is shown in Figures 1A,B.

## 2 Materials and methods

### 2.1 Animals

A total of 72 eight-week-old specific-pathogen-free male C57BL/6J mice, weighing 20 ± 1 g, were used in this experiment. The mice were purchased from SI PEI FU (Beijing) Biotechnology Co., Ltd. (Beijing, China). Animals were kept in a barrier environment with a free diet and water. Environmental conditions were maintained as follows: temperature: 22 ± 2 °C, humidity: 50–60%, noise level: <60 dB, lighting: 12-h light/dark cycle, density: 6 mice per cage. After 7 days of adaptive feeding, the mice were randomly divided into the following groups using random number table method: Sham group (*n* = 16), BCCAO group (*n* = 16), BCCAO + EA group (*n* = 16), BCCAO + EA + THP group (*n* = 8), BCCAO + EA + MLA group (*n* = 8), and BCCAO + Control EA group (*n* = 8) (Figure 1A). This experiment was approved by the Medical and Experimental Animal Ethics Committee of Beijing University of Chinese Medicine on May 27, 2024 (Ethical Approval Number: BUCM-2024052704-2223).

### 2.2 Model preparation

The mouse VaD model was established using BCCAO surgery (Bhatia et al., 2021; Hu et al., 2017). The mice were fasted for 12 h and deprived of water for 4 h before surgery. Anesthesia was induced with 2% isoflurane and maintained with 1.5% isoflurane. The mice were fixed in the supine position. Neck hair was shaved, and the neck skin was cut along the midline to expose bilateral neck musculature. The common carotid arteries (CCAs) and accompanying vagus nerves were carefully separated. A 4-0 suture was looped around each CCA for later use (Figure 1C). The CCAs were clamped with vascular clips for 20 min to block blood flow (Figure 1D). Concurrently, the tail tip was transected 1 cm from the end for bloodletting (15 mL/kg), and hemostasis was achieved by thermal cauterization. After releasing the clips and allowing 10 min of reperfusion, the CCAs were re-clamped for an additional 20 min. After removing the clips and sutures, the mice were observed for 30 min. Then, suture the skin. Core body temperature was maintained at 37 °C throughout the procedure using a thermostatic heating blanket. Penicillin sodium (500,000 IU/kg/day) was injected intraperitoneally for 3 consecutive days after the operation. The mice in the Sham group underwent the same surgical exposure and vessel separation but without artery occlusion or tail bloodletting.

### 2.3 Model evaluation—novel object recognition test

The novel object recognition test was used to evaluate whether the model was qualified (Wang et al., 2025; Zou et al., 2023). An opaque box measuring 40 cm × 40 cm × 40 cm was placed in a dimly lit testing room. Three objects (A, B, and C) were prepared. Objects A and B were identical, while Object C differed from A and B in both color and shape. A video camera was mounted above the box to record the test.

**Figure 2:**
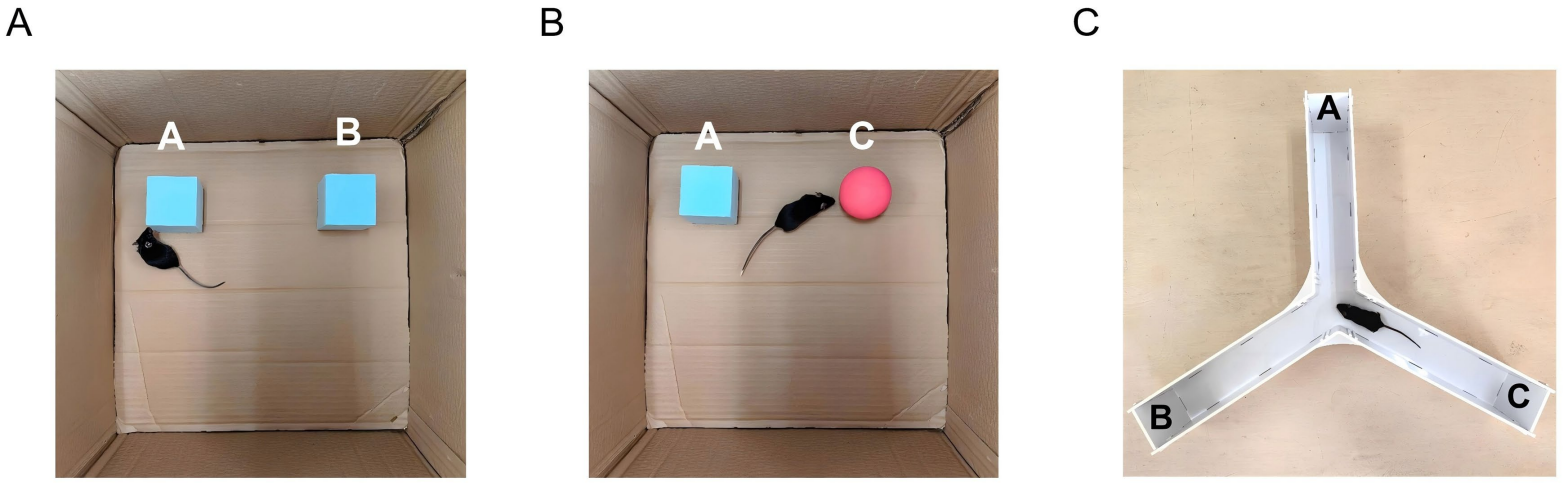
Behavioral tests. **(A,B)** Novel object recognition test. Objects A and B were two identical cubes. Mice were allowed to freely explore the box for 5 min. One hour later, Object B was replaced with Object C, which had a different shape and color. Then, mice were reintroduced into the box. The time spent exploring each object during a subsequent 5-min session was recorded. **(C)** Y-maze test. Mice were allowed to freely explore the maze for 8 min, and the sequence of their entries into each arm was recorded.

First, the mice were placed into the empty box to explore freely for 10 min. Subsequently, Objects A and B were secured within the box, positioned 10 cm from the side walls. After a 24-h interval, each mouse was placed into the box facing away from the objects, equidistant from A and B. The mice were allowed to freely explore the box for 5 min (Figure 2A). One hour later, Object B was replaced with the novel Object C (secured in position), and mice were reintroduced into the box (Figure 2B). The time spent exploring each object (defined as touching the object or sniffing within a 2 cm radius of the object) during a subsequent 5-min session was recorded. The total exploration time of the novel and familiar objects had to exceed 20 s for a valid trial.

After each mouse was removed, feces were cleared, and a spray bottle was used to apply a fine mist of 75% ethanol to eliminate residual odors on the objects and the box. The ethanol was allowed to dry completely before evaluating the next mouse. Discrimination ratio = [Novel object exploration time/(Novel object exploration time + Familiar object exploration time)] × 100%. The discrimination ratio less than 60% indicated successful model establishment.

### 2.4 Electroacupuncture treatment

After the mice regained consciousness on the day of surgery, they were restrained in a prone position using medical adhesive tape. The BCCAO + EA, BCCAO + EA + THP, and BCCAO + EA + MLA groups received EA intervention. We used filiform needles (0.18 × 10 mm, Zhongyan Taihe, Beijing, China) to stimulate Baihui (GV20) acupoint, bilateral Geshu (BL17) acupoints, and bilateral Shenshu (BL23) acupoints. The locations of the acupoints are shown in Figure 1E.

Needles were inserted obliquely at a 5 mm depth from posterior to anterior. For GV20 acupoint, the needle reached the subgaleal space. For BL17 and BL23 acupoints, needles were inserted into the muscle layer. The negative electrode of the EA instrument (SDZ-II, Hwato, Suzhou, China) was connected to the GV20 needle. The positive electrode was clipped to a small piece of saline-moistened gauze wrapped around a forelimb. The ipsilateral BL17 needle (positive electrode) was connected to the BL23 needle (negative electrode). Continuous wave was used with a frequency of 2 Hz and an intensity of 1 mA for 10 min each session, once a day for 7 days. For the BCCAO + Control EA group, needles were inserted bilaterally below the costal margin and 5 mm from the ilium (non-acupoint) (Liu et al., 2023). The left side was connected to the cathode, and the right side to the anode. The acupuncture method, EA parameters, and treatment duration were identical to those used in the BCCAO + EA group. The Sham group and BCCAO group were restrained in the same position for 10 min daily during the corresponding period.

For the BCCAO + EA + THP group, THP, the m-1AChR antagonist, was intraperitoneally injected 30 min before daily EA treatment (1.5 mg/kg). For the BCCAO + EA + MLA group, MLA, the α-7-nAChR antagonist, was intraperitoneally injected 30 min before daily EA treatment (5 mg/kg). The injection volume was 5 mL/kg in both groups. The mice in Sham group, BCCAO group, and BCCAO + EA group received intraperitoneal injections of 5 mL/kg normal saline at the same time every day.

### 2.5 Novel object recognition test

Following 7 days of EA treatment, recognition function in mice was evaluated using the novel object recognition test. The test procedure was described in “Section 2.3 Model evaluation—Novel Object Recognition test”. The discrimination index (DI) was calculated using the formula (Zhao et al., 2023): DI = (Novel object exploration time − Familiar object exploration time)/(Novel object exploration time + Familiar object exploration time). A higher DI indicates a stronger preference for the novel object and better object recognition memory ability in the mice.

### 2.6 Y-maze test

The Y-maze test was performed according to previously reported methods (Wei et al., 2024). The Y-maze consisted of three symmetrical arms (A, B, and C) made of white plastic, arranged at 120° angles to each other. Each arm measured 30 cm in length, 6 cm in width, and 15 cm in height. A video camera was positioned above the maze to record the movement trajectories of the mice.

The mouse was placed at the center of the maze and allowed to explore freely for 8 min (Figure 2C). An arm entry was recorded when all four paws of the mouse entered an arm. A spontaneous alternation was counted when the mouse consecutively entered three different arms. For example, if the sequence of arm entries is ACBCACBA, the total number of arm entries is 8, and the number of spontaneous alternations is 4 (ACB, BCA, ACB, CAB). Spontaneous alternation rate = [Number of spontaneous alternations/(Total number of arm entries − 2)] × 100%. A higher spontaneous alternation rate indicates better spatial working memory. After each test, feces were removed, and the Y-maze was cleaned with 75% ethanol.

### 2.7 Microscopic imaging of cerebral microvessels

After 7 days of EA intervention, cerebral microvessels of mice were observed. Mice were fasted for 12 h and water-deprived for 4 h before detection. Anesthesia was maintained with isoflurane. Mice were secured in the prone position. After shaving the scalp hair, a midline incision was made to expose the skull. A 4 × 4 mm cranial window was created using a handheld micro-drill (SRONG 102LN, Sitaijie, Fuzhou, China) 1 mm posterior to the coronal suture and 1 mm to the right of the sagittal suture. Continuous drops of 37 °C normal saline were administered during drilling to prevent thermal injury.

The pia mater and tiny hairs were carefully removed under a stereo microscope (SZ2-ILST, Olympus, Tokyo, Japan). Microvessels within the window were visualized using the SMC1 ultra-high-speed dynamic microcirculation imaging system (Gene&I-SMCI, Gene&I, United States). Small branches of the anterior cerebral artery and superior cerebral vein were observed, selected based on the following criteria: arterial diameters between 10–30 μm, venular diameters between 30–50 μm, unbranched single vessels with minimal curvature, and length approximately 200 μm. Microvascular images were continuously recorded with a high-speed camera (FASTCAM-ultima APX, photron, Tokyo, Japan) at 500 frames per second for 10 s. After recordings, mice were sacrificed for brain tissue collection, which was then subjected to the tests outlined in the following sections. Vessel diameters were measured using ImageJ software (NIH, MD, United States) with triplicate measurements averaged per vessel. The RBC flow velocity was measured by slow playback video. RBC flow velocity = RBC movement length/time. To normalize the data, both vascular diameter and RBC velocity were presented as ratios relative to the average of the Sham group.

### 2.8 Immunofluorescence staining

After 7 days of EA intervention, mice were anesthetized and transcardially perfused with normal saline, followed by 4% paraformaldehyde. Brains were extracted and fixed in 4% paraformaldehyde for 24 h, then dehydrated through a graded ethanol series. After paraffin embedding, brains were sectioned coronally at 4 μm thickness. Sections (interaural 4.54 mm, bregma 0.74 mm) were blocked with 3% bovine serum albumin for 30 min. Subsequently, sections were incubated overnight with primary antibody (anti-c-Fos, 1:500, Servicebio, Wuhan, China). Following washes, sections were incubated with fluorescent secondary antibody (CY3-conjugated goat anti-mouse IgG, 1:300, Servicebio, Wuhan, China). Images were acquired using the Eclipse C1 fluorescence microscope (Nikon, Japan). At a magnification of 400×, three fields of view were randomly selected within the nucleus of the horizontal limb of the diagonal band (hDB) in the basal forebrain per tissue section for analysis [refer to the anatomical atlas (Paxinos and Franklin, 2012)], and the results were averaged. The percentage of c-Fos positive cells and mean fluorescence intensity were quantified using ImageJ software (NIH, MD, United States). The images were split into red and blue channels (8-bit), and thresholding was performed using the default algorithm. Non-specific staining in the background was removed using the binary option. Then, the overlap between the red and blue channels was taken to count the number of positive cells. The percentage of c-Fos positive cells was calculated as: (Number of cells in the overlap of red and blue channels/Number of cells in the blue channel) × 100%. The mean fluorescence intensity was defined as the mean gray value of the red channel.

### 2.9 Biochemical method detection

Following 7 days of EA intervention, the mice were anesthetized and then sacrificed by cervical dislocation. Brains were rapidly dissected on an ice tray, and the basal forebrains were separated. ACh content (μg/mg prot) in the basal forebrain was quantified according to the instructions of the Acetylcholine Assay Kit (NJJC Bio, Nanjing, China).

### 2.10 Western blot

Frontal cortex tissue was isolated and homogenized in lysis buffer at a 1:10 tissue-to-buffer ratio to obtain total protein lysates. Proteins were separated by SDS-PAGE and electrophoretically transferred onto PVDF membranes (Servicebio, Wuhan, China). Membranes were blocked with 5% skim milk for 30 min, followed by incubation with primary antibodies at 4 °C overnight. The primary antibodies included anti-m-1AChR (1:1,000, Biodragon, Beijing, China), anti-α-7-nAChR (1:1,000, Abcam, Cambridge, UK), anti-eNOS (1:3,000, Servicebio, Wuhan, China), and anti-ACTIN (1:3,000, Servicebio, Wuhan, China). After washing three times, membranes were incubated with the secondary antibody (HRP-conjugated Goat Anti-Rabbit IgG (H + L), 1:3,000, Servicebio, Wuhan, China) for 30 min at room temperature. After washing three times, membranes were immersed in ECL substrate for 1 min. Chemiluminescent signals were detected using the SCG-W3000 Imaging System (Servicebio, Wuhan, China). The original images were analyzed with AIWBwell^™^ software (Servicebio, Wuhan, China).

### 2.11 Quantitative real-time polymerase chain reaction

Total RNA was extracted from frontal cortex tissue. It was then reverse transcribed into cDNA using a reverse transcription kit (Servicebio, Wuhan, China). Quantitative real-time polymerase chain reaction (qRT-PCR) amplification was performed on a CFX Connect^™^ Real-Time Polymerase Chain Reaction Detection System (Bio-Rad, CA, United States) with the following protocol: initial denaturation at 95 °C for 30 s, 40 cycles were performed for denaturation at 95 °C for 15 s and annealing at 60 °C for 30 s (Camacho et al., 2023). Results were analyzed using the “ $2^{-\Delta \Delta \mathrm{CT}}$ method.” The GAPDH primer pair was used as the internal control. The primer sequences used were as follows:

GAPDH forward: 5′-CCTCGTCCCGTAGACAAAATG-3′.
GAPDH reverse: 5′-TGAGGTCAATGAAGGGGTCGT-3′.
eNOS forward: 5′-CAATCTTCGTTCAGCCATCACAG-3′.
eNOS reverse: 5′-GGAGCCATCCTGCTGCCTAT-3′.
m-1AChR forward: 5′-TCAGTCCCAACATCACCGTCTT-3′.
m-1AChR reverse: 5′-AGGCTCAGCAGGAAGTAGTTGTT-3′.
α-7-nAChR forward: 5′-TGGAGGAGGTCCGCTACATC-3′.
α-7-nAChR reverse: 5′-AGTTTGGGGCTGACATGAGGA-3′.

### 2.12 Griess-colorimetric method detection

The content of NO in frontal cortex was measured using a Nitrate Reductase (NR) Activity Assay Kit (Solarbio, Beijing, China) according to the instructions. Absorbance at 540 nm was determined using an Epoch microplate spectrophotometer (BioTek, VT, United States). NR activity was calculated (U/mg prot), and higher NR activity indicated higher NO content.

### 2.13 Statistical analysis

All data were analyzed using SPSS software (version 25, IBM, Chicago, IL, United States), with results presented as mean ± standard error of the mean (SEM). Normality was first assessed using the Shapiro–Wilk test. If the distribution was normal, one-way analysis of variance (ANOVA) was used for multiple group comparisons. When the variance was homogeneous, pairwise comparisons were conducted using the least significant difference (LSD) method. In cases of unequal variance, the Games–Howell method was used for pairwise comparisons. Non-normally distributed data were analyzed using the nonparametric Kruskal–Wallis test, and pairwise comparisons between groups were corrected using the Bonferroni method. The *p*-value less than 0.05 was considered the threshold for statistical significance. Bar graphs were generated using Prism (version 9.5, GraphPad Software, San Diego, CA, United States).

## 3 Result

### 3.1 EA alleviated the cognitive impairment in VaD mice

**Figure 3:**
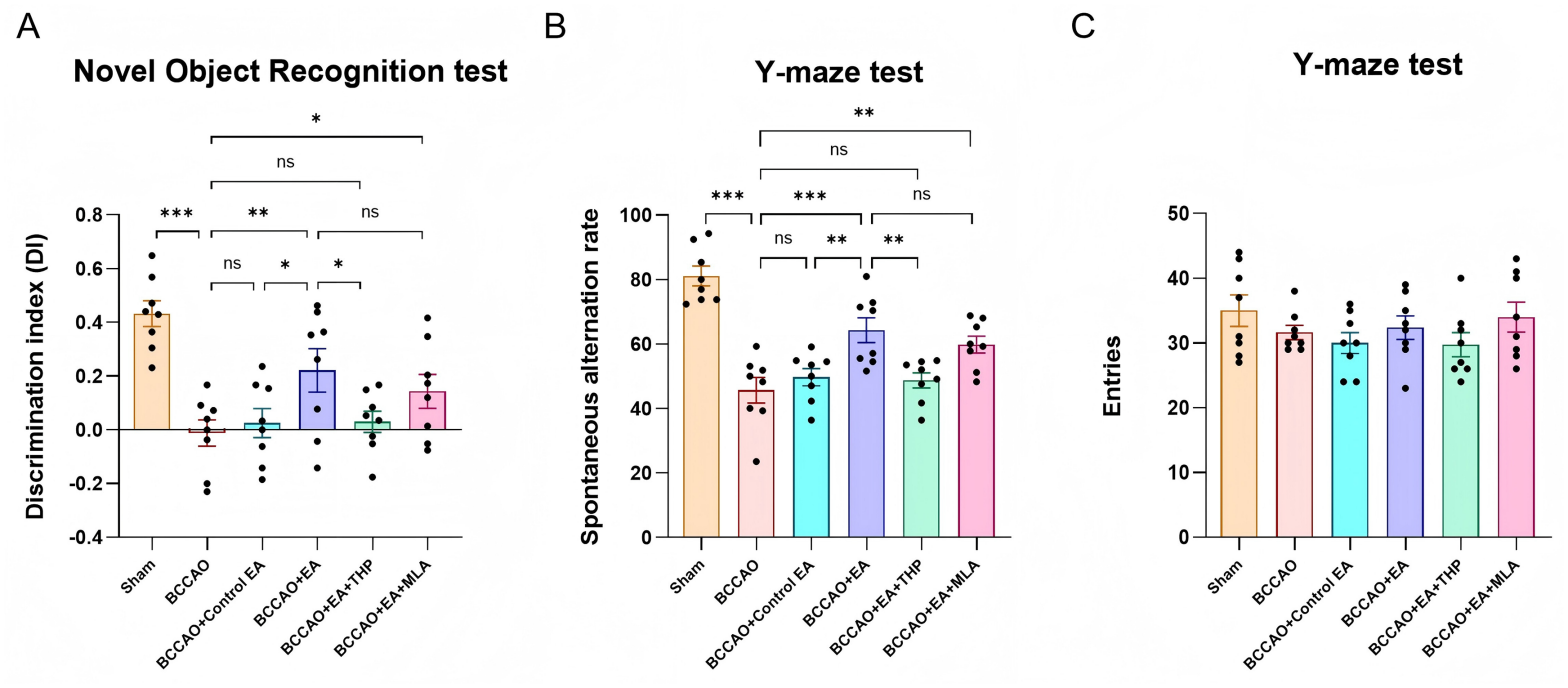
**(A)** The novel object recognition test results of mice in each group were compared. **(B)** The Y-maze test results of mice in each group were compared. Data were presented as mean ± SEM (*n* = 8 per group). One-way ANOVA followed by the least-significant difference test. ^ns^*p* > 0.05, ^*^p < 0.05, ^**^p < 0.01, and ^***^p < 0.001. **(C)** The total number of arm entries for each mouse in Y-maze test was presented. VaD, vascular dementia; EA, electroacupuncture; BCCAO, bilateral common carotid artery occlusion; THP, trihexyphenidyl hydrochloride; MLA, methyllycaconitine citrate.

After 7 days of EA treatment, the recognition memory ability of mice was assessed using novel object recognition test, and the spatial working memory ability was evaluated by Y-maze test. Both the DI (*F*_5,42_ = 8.778, *p* < 0.001) (Figure 3A) and the spontaneous alternation rate (*F*_5,42_ = 18.009, *p* < 0.001) (Figure 3B) in the BCCAO group were lower compared to the Sham group, while EA treatment significantly improved these two indicators relative to the BCCAO group (*p* < 0.01, *p* < 0.001). Figure 3C shows the total number of arm entries for each mouse in Y-maze test. The activity levels of mice in each group were similar (*H* = 4.341, *p* > 0.05). These results demonstrated that EA alleviated the cognitive impairment in VaD mice, specifically improving recognition memory and spatial working memory.

Compared with the BCCAO group, non-acupoint EA did not improve the DI (*p* > 0.05) or the spontaneous alternation rate (*p* < 0.05). Both of these indices were significantly lower in the BCCAO + Control EA group than in the BCCAO + EA group (*p* < 0.05, *p* < 0.01). These results indicated that non-acupoint EA did not ameliorate memory impairment in VaD mice.

The DI (*p* < 0.05) and spontaneous alternation rate (*p* < 0.01) in the BCCAO + EA + THP group were lower than those in the BCCAO + EA group, but showed no significant difference versus the BCCAO group (*p* > 0.05, *p* > 0.05). These two indicators in the BCCAO + EA + MLA group were not significantly different from those in the BCCAO + EA group (*p* > 0.05, *p* > 0.05), but were higher than those in the BCCAO group (*p* < 0.05, *p* < 0.01). The results showed that THP almost reversed the therapeutic effect of EA on memory function in VaD mice, whereas MLA failed to reverse this effect.

### 3.2 EA dilated the leptomeningeal arterioles in VaD mice

**Figure 4:**
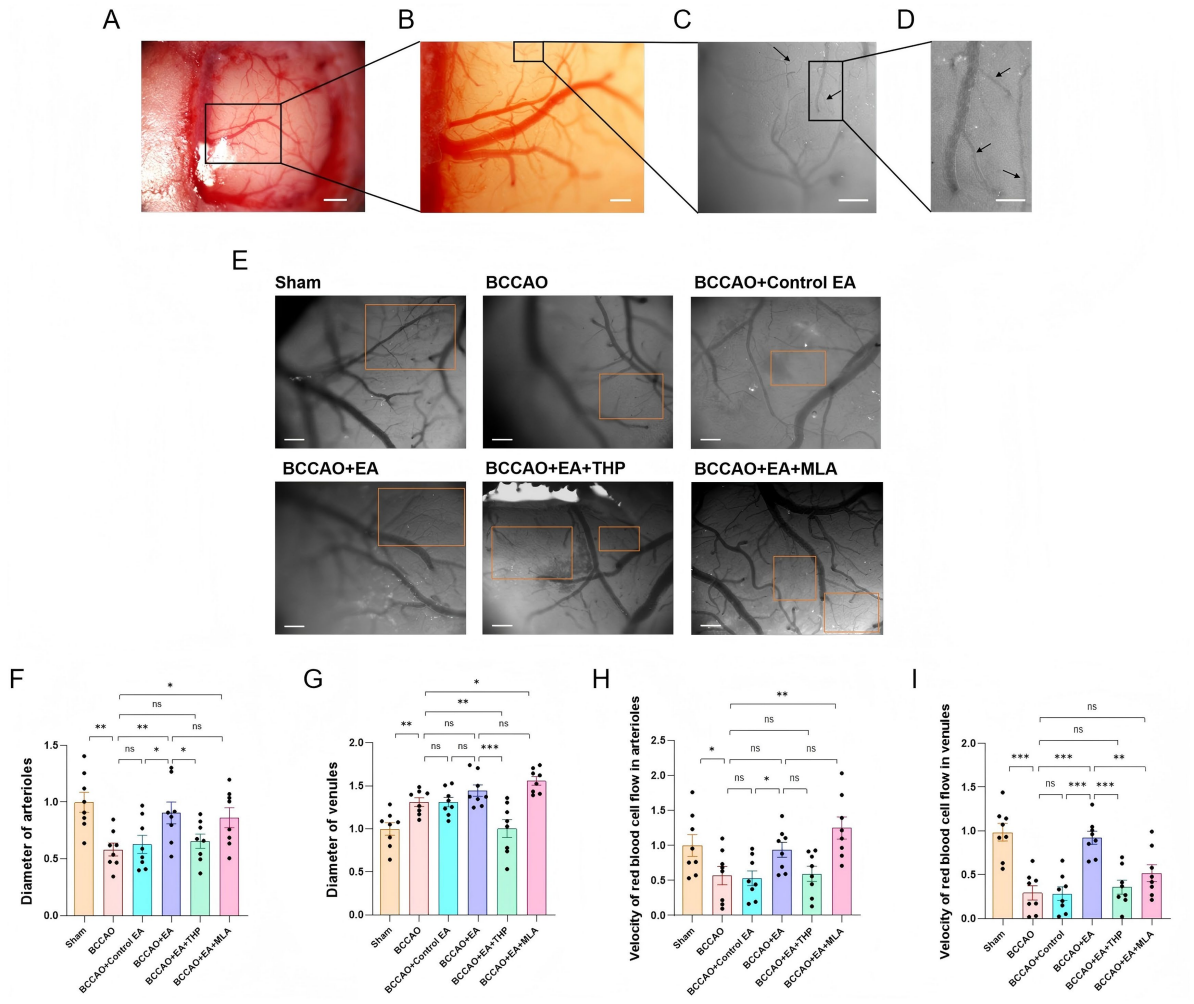
EA dilated the leptomeningeal arterioles in VaD mice. **(A)** Leptomeningeal vessels in the cranial window were visualized under stereo microscopy. Bar = 1 mm. **(B)** Under a 5× objective lens, it could be seen that the multilevel vascular branches formed an extensive microvascular network. Bar = 500 μm. **(C)** Visible terminal branches were dynamically monitored using the ultra-high-speed dynamic microcirculation imaging system under a 10× objective lens. Arterioles and venules could be distinguished by the direction of blood flow. Bar = 200 μm. **(D)** By magnifying the local area, the terminal branches can be seen more clearly. Arrows indicate microvessels meeting the observation criteria. Bar = 100 μm. **(E)** Representative images of cerebral microvessel microscopic imaging of mice in each group. The orange boxes indicate the suitable observation areas. Bar = 200 μm. **(F,G)** The diameters of leptomeningeal microvessels in each group were compared. **(H,I)** The red blood cell flow velocity in leptomeningeal microvessels in each group was compared. Data were presented as mean ± SEM (*n* = 8 per group). One-way ANOVA followed by the least-significant difference test. ^ns^*p* > 0.05, ^*^p < 0.05, ^**^p < 0.01, and ^***^p < 0.001. VaD, vascular dementia; EA, electroacupuncture; BCCAO, bilateral common carotid artery occlusion; THP, trihexyphenidyl hydrochloride; MLA, methyllycaconitine citrate.

After 7 days of EA intervention, cerebral microvessels of mice were observed. The pial microvessels penetrate the brain parenchyma to supply the cerebral tissue. Therefore, the state of the pial microcirculation can reflect CBF. Following cranial window creation, leptomeningeal vessels were visualized under stereo microscopy (Figure 4A). Under a 5× objective lens, multilevel vascular branches formed an extensive microvascular network (Figure 4B). Visible terminal branches were observed under a 10× objective lens (Figures 4C,D). Using the ultra-high-speed dynamic microcirculation imaging system, RBC flow direction was dynamically monitored, and the arterioles and venules could be distinguished. Microscopic observation revealed relatively dense microvasculature in the Sham group (Figure 4E). In contrast, the BCCAO group exhibited attenuated vessel diameter and reduced microvascular density. The BCCAO + EA group demonstrated increased vessel diameter compared to the BCCAO group. As for the two inhibitor groups, the microvessels in the BCCAO + EA + THP group were relatively narrow, while those in the BCCAO + EA + MLA group were relatively wide.

All parameters were normalized by calculating ratios relative to the mean values of the Sham group for standardized processing. Compared with the Sham group, the arteriolar diameters were reduced (*F*_5,42_ = 4.662, *p* < 0.01) (Figure 4F), the venular diameters were increased (*F*_5,42_ = 11.001, *p* < 0.01) (Figure 4G), and the RBC flow velocity in both arterioles (*F*_5,42_ = 5.088, *p* < 0.05) and venules (*F*_5,42_ = 13.729, *p* < 0.001) were decreased in the BCCAO group (Figures 4H,I). Compared with the BCCAO group, the arteriolar diameters (*p* < 0.01) and the RBC flow velocity of the venules (*p* < 0.001) in the BCCAO + EA group were significantly increased. The venular diameters and the arteriolar RBC flow velocity had an upward trend, but there was no significant difference (*p* > 0.05, *p* > 0.05). These findings demonstrated that EA dilated leptomeningeal arterioles and accelerated venular blood flow velocity.

Compared to the BCCAO group, non-acupoint EA failed to produce a significant impact on the aforementioned four parameters (*p* > 0.05) (Figures 4F–I). In comparison with the BCCAO + EA group, the BCCAO + Control EA group exhibited significantly smaller arteriolar diameters (*p* < 0.05) and significantly lower RBC velocities in both arterioles (*p* < 0.05) and venules (*p* < 0.001). These results indicated that non-acupoint EA did not regulate leptomeningeal arterioles and venules in VaD mice.

Compared with the BCCAO + EA group, the arteriolar diameter (*p* < 0.05), venular diameter (*p* < 0.001), and venular RBC flow velocity (*p* < 0.001) in the BCCAO + EA + THP group were decreased, while there was no significant difference in arteriolar RBC flow velocity between the two groups (*p* > 0.05) (Figures 4F–I). Compared with the BCCAO + EA group, the BCCAO + EA + MLA group showed no significant difference in arteriolar diameter (*p* > 0.05), venular diameter (*p* > 0.05), or arteriolar RBC flow velocity (*p* > 0.05), while the venular RBC flow velocity was decreased (*p* < 0.01). The results indicated that THP reversed the dilatational effect of EA on leptomeningeal arterioles and the acceleration of venular RBC flow velocity in VaD mice, whereas MLA failed to reverse these effects.

Since the effect of acupoint therapy had been confirmed, the BCCAO + Control EA group would not be set up for subsequent tests.

### 3.3 EA increased the activity of nerve cells in the hDB nucleus region in basal forebrain of VaD mice

c-Fos, an immediate early gene, exhibits low basal expression in the nervous system under physiological conditions. However, its protein product undergoes rapid transcriptional upregulation within minutes following various physical/chemical stimuli, making c-Fos immunoreactivity a widely used marker of neuronal activation (Cruz-Mendoza et al., 2022). As a key mediator regulating NVC, ACh in the neocortex is entirely derived from cholinergic neurons in the basal forebrain. The basal forebrain is located in the ventral part of the forebrain and comprises multiple nucleus cluster. Among them, cholinergic neurons in the hDB nucleus project extensively to the cerebral cortex and play a crucial role in cognitive function (Wu et al., 2025). Therefore, the level of c-Fos protein in the hDB nucleus region indirectly reflects the activity of ACh projections to the cerebral cortex.

**Figure 5:**
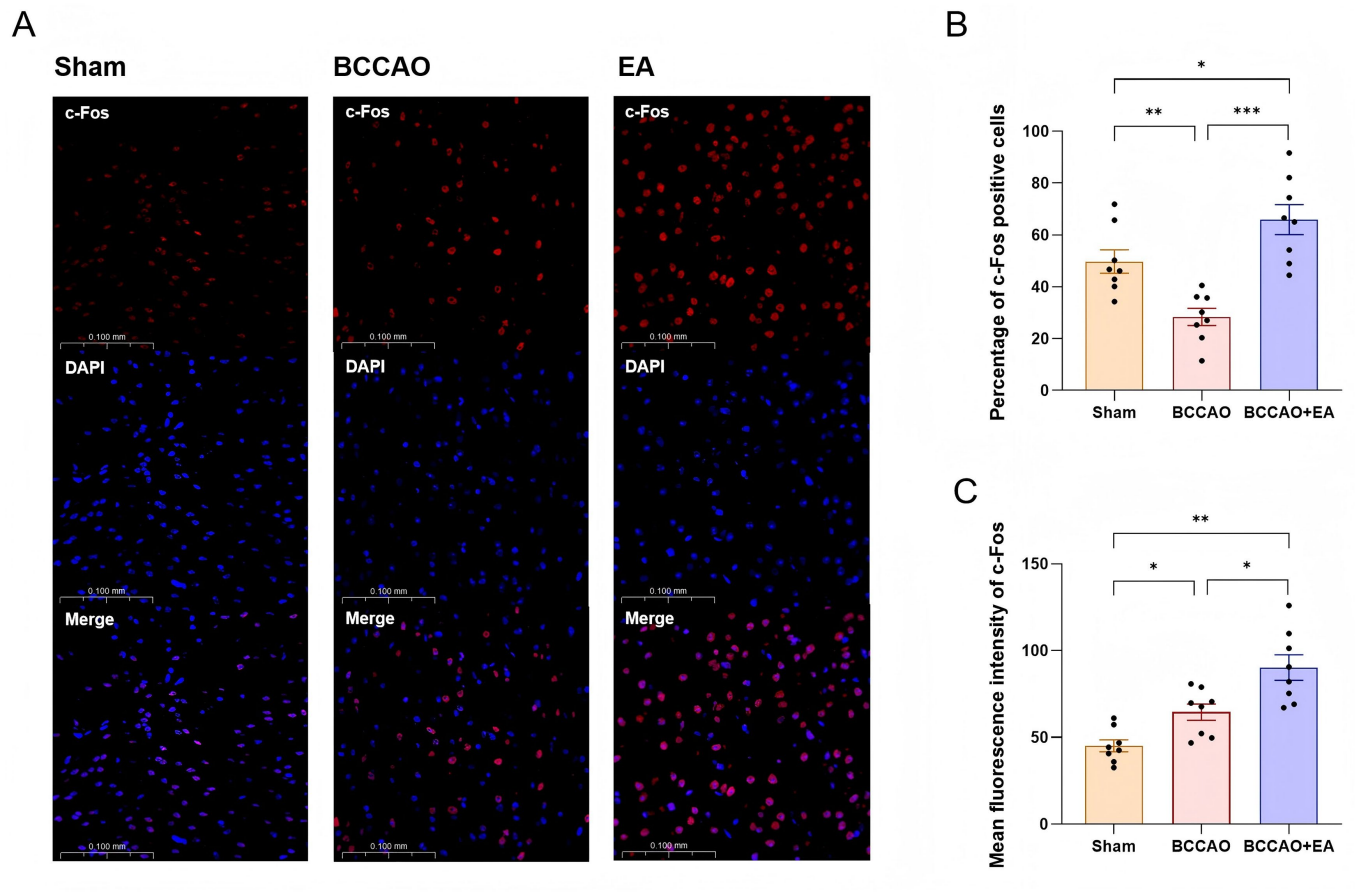
EA increased the activity of nerve cells in the hDB nucleus region in basal forebrain of VaD mice. **(A)** Representative immunofluorescence images of c-Fos protein in the hDB nucleus region in basal forebrain of each group. **(B,C)** The percentage of c-Fos positive cells and the mean fluorescence intensity of c-Fos protein were semi-quantitatively analyzed. Data were presented as mean ± SEM (*n* = 8 per group). One-way ANOVA followed by the least-significant difference test or the Games–Howell test. ^*^p < 0.05, ^**^p < 0.01, and ^***^p < 0.001. VaD, vascular dementia; EA, electroacupuncture; hDB, horizontal limb of the diagonal band; BCCAO, bilateral common carotid artery occlusion.

Representative immunofluorescence images of c-Fos protein in the basal forebrain across groups after 7 days of treatment are shown in Figure 5A. Compared with the Sham group, the percentage of c-Fos protein positive cells in the BCCAO group decreased (*F*_2,21_ = 16.314, *p* < 0.01) (Figure 5B), while the mean fluorescence intensity of c-Fos protein increased (*F*_2,21_ = 17.264, *p* < 0.05) (Figure 5C). EA treatment significantly elevated both the percentage of c-Fos protein positive cells (*p* < 0.001) and the mean fluorescence intensity of c-Fos protein (*p* < 0.05). These two indicators in the BCCAO + EA group were even higher than those in the Sham group (*p* < 0.05, *p* < 0.01). These results indicated that EA increased the activity of nerve cells in the hDB nucleus region in basal forebrain of VaD mice and enhanced c-Fos protein expression.

### 3.4 EA up-regulated the ACh/NO pathway in the cholinergic vasodilation system in VaD mice

**Figure 6:**
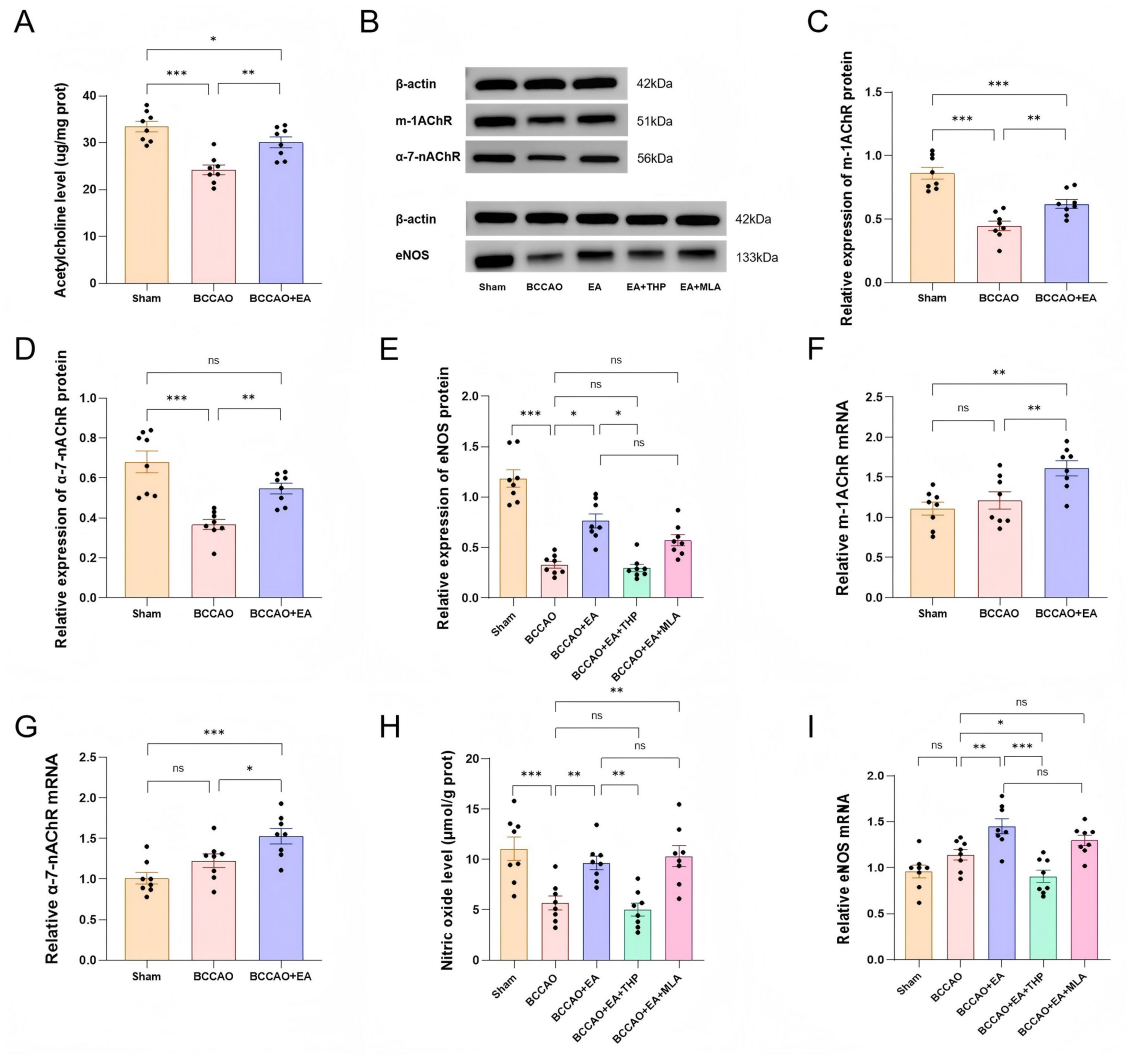
EA up-regulated the ACh/NO pathway within the cholinergic vasodilation system in VaD mice. **(A)** The levels of acetylcholine in the basal forebrain of mice in each group were quantitatively analyzed. **(B)** Representative immunoblot bands of m-1AChR, α-7-nAChR, and eNOS proteins. **(C–E)** The protein content of m-1AChR, α-7-nAChR, and eNOS in the frontal cortex of VaD mice was semi-quantitatively analyzed. **(F–H)** Relative levels of m-1AChR mRNA, α-7-nAChR mRNA, and eNOS mRNA in each group were compared. **(I)** The levels of nitric oxide in the frontal cortex of mice in each group were quantitatively analyzed. Data were presented as mean ± SEM (*n* = 8 per group). One-way ANOVA followed by the least-significant difference test or the Kruskal–Wallis test. ^ns^*p* > 0.05, ^*^p < 0.05, ^**^p < 0.01, ^***^p < 0.001. m-1AChR, muscarinic acetylcholine receptor 1; α-7-nAChR, α-7-nicotinic acetylcholine receptor; eNOS, endothelial nitric oxide synthase; mRNA, messenger ribonucleic acid; VaD, vascular dementia; EA, electroacupuncture; BCCAO, bilateral common carotid artery occlusion; THP, trihexyphenidyl hydrochloride; MLA, methyllycaconitine citrate.

Studies have found that ACh binding to acetylcholine receptors can induce the activation of eNOS, thereby increasing NO levels, which leads to vasodilation and an increase in CBF (Nizari et al., 2021). After 7 days of treatment, the ACh level in basal forebrain was decreased in the BCCAO group compared to the Sham group (*F*_2,21_ = 17.862, *p* < 0.001). EA treatment significantly increased ACh content versus BCCAO group (*p* < 0.01), though the levels remained below the Sham group (*p* < 0.05) (Figure 6A).

The frontal cortex is closely related to cognitive functions, so we measured the relevant indicators of the frontal cortex. Figure 6B shows representative immunoblot bands of m-1AChR, α-7-nAChR, eNOS, and the loading control β-actin in the frontal cortex of each group after 7 days of treatment. Compared with the Sham group, the expression of m-1AChR (*F*_2,21_ = 27.103), α-7-nAChR (*H* = 16.150), and eNOS (*H* = 32.259) in the BCCAO group was decreased (*p* < 0.001) (Figures 6C–E). EA treatment significantly increased the expression of m-1AChR (*p* < 0.01), α-7-nAChR (*p* < 0.01), and eNOS (*p* < 0.05) versus BCCAO group. The expression level of m-1AChR in the BCCAO + EA group was still lower than that in the Sham group (*p* < 0.001), while there was no significant difference in the expression of α-7-nAChR between the two groups (*p* > 0.05).

The qRT-PCR results of m-1AChR, α-7-nAChR, and eNOS in frontal cortex showed similar trends (Figures 6F–H). The BCCAO group exhibited no significant differences versus the Sham group in m-1AChR mRNA (*F*_2,21_ = 7.896), α-7-nAChR mRNA (*F*_2,21_ = 9.724), and eNOS mRNA (*F*_4,35_ = 11.699) (*p* > 0.05). The above three indicators in the BCCAO + EA group were significantly higher than those in the BCCAO group (*p* < 0.01, *p* < 0.05, *p* < 0.01).

Compared with the Sham group, the level of NO in the frontal cortex of the BCCAO group was decreased (*F*_4,35_ = 10.297, *p* < 0.001). EA treatment significantly elevated the NO level versus BCCAO group (*p* < 0.01) (Figure 6I).

In summary, EA up-regulated the ACh/NO pathway within the cholinergic vasodilation system, increased the content of ACh in the basal forebrain, promoted the expression of m-1AChR, α-7-nAChR, and eNOS in the frontal cortex, and elevated the level of NO in the frontal cortex in VaD mice.

As for the two inhibitor groups, the results of Western blot (WB) showed that eNOS protein expression in the BCCAO + EA + THP group was decreased compared with the BCCAO + EA group (*p* < 0.05), while showing no significant difference from the BCCAO group (*p* > 0.05) (Figure 6E). The results of PCR indicated that the relative quantity of eNOS mRNA in the BCCAO + EA + THP group was decreased compared with the BCCAO + EA group (*p* < 0.001), and was also lower than that in the BCCAO group (*p* < 0.05) (Figure 6H). However, both eNOS protein expression and the relative quantity of eNOS mRNA in the BCCAO + EA + MLA group showed no significant differences compared with the BCCAO + EA group and the BCCAO group (*p* > 0.05). The NO content in the BCCAO + EA + THP group was decreased compared with the BCCAO + EA group (*p* < 0.01), with no significant difference from the BCCAO group (*p* > 0.05) (Figure 6I). The NO content in the BCCAO + EA + MLA group showed no significant difference compared with the BCCAO + EA group (*p* > 0.05), but was higher than that in the BCCAO group (*p* < 0.01). The results suggested that THP reversed the up-regulation effects of EA on eNOS and NO in the frontal cortex of VaD mice, while MLA could not reverse these effects.

These findings demonstrated that THP, but not MLA, partially reversed the effect of EA on the ACh/NO pathway within the cholinergic vasodilation system in VaD mice.

## 4 Discussion

In this study, we found that EA alleviated cognitive impairment in VaD mice by regulating cholinergic vasodilation system, which involves activating cholinergic neurons, increasing ACh release in basal forebrain, up-regulating m-1AChR in frontal cortex, promoting eNOS expression, enhancing NO release, and promoting cerebral arterioles dilation.

Blood vessels and nerves at various levels exhibit structural and functional connections throughout the body. They constitute the neurovascular units and rely on NVC to achieve interactive functions. The specific process of NVC involves astrocytes establishing dynamic connections between neurons and blood vessels through their foot processes. Vasoactive substances such as NO, prostaglandins, arachidonic acid, thromboxane, and neuropeptides, mediate the stimulation to vascular smooth muscle cells (Guerra et al., 2018; Moccia et al., 2022). The stimulation triggers changes in membrane potential and intracellular Ca^2+^ concentration, subsequently causing contraction or relaxation of smooth muscle (Longden et al., 2016). The close coordination between neural activity and hemodynamics maintains normal physiological brain function.

In cerebral small vessel disease, arteriolar smooth muscle cells become stiff and proliferative, reducing vasodilation and impairing NVC (van Veluw et al., 2022; Zanon et al., 2021). Chronic cerebral hypoperfusion reduces CBF below physiological needs. This fails to sustain normal metabolic processes and induces VaD. Therefore, regulating microvascular dilation and improving cerebral perfusion are necessary for the prevention and treatment of VaD.

We established the mouse VaD model using BCCAO surgery in this experiment. For EA treatment, we selected the acupoints GV20, BL17, and BL23. These three acupoints are commonly used in clinical traditional Chinese medicine for treating VaD. Studies have confirmed that EA at acupoints including GV20 and BL23 can improve learning and memory in VaD rats (Zhu et al., 2012). Previous research from our team found that administering EA at GV20, BL17, and BL23 for 7 consecutive days improved neurological function and spatial memory ability, while inhibiting hippocampal neuronal apoptosis in VaD mice (Liu et al., 2023). Bibliometric research also indicates that GV20 is the most frequently used acupoint for treating cognitive impairment with acupuncture. Therefore, we chose the acupoint combination of GV20, BL17, and BL23.

A review of the literature indicated that in animal studies of EA for cardiovascular and cerebrovascular diseases, a stimulation intensity of 1 mA is predominantly used, with low frequencies (2 Hz, 10 Hz) (Zeng et al., 2024). Research on standardizing acupuncture parameters for VaD rats recommends that the needle be retained for at least 10 min per session, with treatment administered once daily (Yang et al., 2020). Our pilot study showed that 1 mA for 10 min daily over 7 days was effective and well-tolerated. Increasing the intensity or prolonging the daily stimulation time resulted in noticeable agitation in the mice. In summary, our final EA parameters were set to 1 mA, 2 Hz, 10 min per day, for 7 consecutive days.

The behavioral test results demonstrated that EA treatment improved recognition memory and spatial working memory abilities of VaD mice. Previous research teams employed similar EA parameters and acupoint selection to treat VaD, reaching similar conclusions as our experiment (Lin and Hsieh, 2010; Zhu et al., 2012).

We used the ultra-high-speed dynamic microcirculation imaging system to observe the leptomeningeal microvessels of mice in each group. Leptomeningeal microvessels penetrate deep into the brain parenchyma and supply nutrients to brain tissue. Therefore, the condition of leptomeningeal microcirculation can reflect cerebral microcirculation. The results showed that 7 days after modeling, the arterioles of VaD mice were in a contracted state, and RBC flow velocity was slower compared to the Sham group, leading to hypoperfusion. EA stimulation significantly dilated the arterioles, reduced the resistance in the arteries, and correspondingly accelerated the flow velocity. Arterioles serve as the primary regulators of blood flow, while venules gradually dilate in response to reduced upstream resistance (Tajima et al., 2014). In this experiment, venular diameter in the BCCAO group was significantly larger than that in the Sham group, RBC flow velocity was decreased, and even intermittent blood flow and blood stasis were observed. This may result from post-ischemic platelet aggregation, leukocyte entrapment, and microthrombi causing venous congestion. Furthermore, venules lack smooth muscle and have poor elasticity. Thus, they dilated passively. After EA stimulation, venular RBC flow velocity significantly increased.

To validate the efficacy of the selected acupoints, we established the BCCAO + Control EA group. The results showed that non-acupoint EA failed to improve recognition memory or spatial working memory in VaD mice, which is consistent with previous findings from our research team (Liu et al., 2023). We further examined the pial microvessels in the BCCAO + Control EA group and found that non-acupoint EA produced no significant effect on the pial microvessels of VaD mice. These findings indicate that appropriate acupoints are necessary for EA treatment in VaD mice. As the therapeutic effect of acupoint stimulation had been well established, subsequent experiments did not include the BCCAO + Control EA group.

ACh is an important mediator of the NVC effect. It induces eNOS activation by binding to acetylcholine receptors, followed by NO release from endothelial cells to induce vasodilation. Acetylcholine receptors are divided into mAChRs and nAChRs. mAChR subtypes (m-1, m-3, m-5) mediate Ca^2+^ release from the endoplasmic reticulum, activating eNOS (Fleming, 2010; Radu et al., 2017). m-1AChR is the most abundant subtype in the neocortex. It is associated with memory processes involving cortical-hippocampal interactions and plays a role in memory consolidation (Ztaou and Amalric, 2019). It has been found that an mAChR blocker (atropine) can eliminate the enhancement effect of EA on cerebral perfusion in mice with cerebral ischemia (Kim et al., 2013). nAChRs are classified into muscle-type and neuronal-type. Neuronal-type nAChRs are widely distributed throughout the human brain and function at presynaptic membranes to regulate the release of several central neurotransmitters. Among them, the α-7-nAChR subtype plays a critical role in the cholinergic anti-inflammatory pathway after cerebral ischemia (Aguado et al., 2023; Duris et al., 2017). However, whether it mediates cortical arteriolar dilation remains controversial (Cheng et al., 2011; Kim et al., 2013; Lee et al., 2011; Nakajima et al., 2003). Therefore, we selected THP (m-1AChR antagonist) and MLA (α-7-nAChR antagonist) as inhibitors for experimental observation.

The performance of BCCAO + EA + THP group mice in behavioral tests was significantly worse than that of the BCCAO + EA group, indicating that THP reversed the improvement effect of EA on memory function in VaD mice. However, there was no statistical difference in performance between the BCCAO + EA + MLA group and the BCCAO + EA group. The result demonstrated that m-1AChR plays an important role in the process of EA improving memory function in VaD mice, while α-7-nAChR may not be involved in this process.

The arteriolar diameter in the BCCAO + EA + THP group was smaller than that in the BCCAO + EA group, which may be because THP antagonized the binding of ACh to m-1AChR, hindered NO generation, and affected NO-mediated vasodilation. There was no significant difference in arteriolar RBC flow velocity between the BCCAO + EA + THP group and the BCCAO + EA group, which may be because other subtypes of mAChRs or nAChRs exerted partial effects, maintaining the signal transduction of the ACh/NO pathway to a certain extent (Guerra et al., 2018). The venular diameter in the BCCAO + EA + THP group was smaller than that in the BCCAO + EA group, and RBC flow velocity was slower. On one hand, THP may have hindered the vasodilatory effect of NO. On the other hand, venules contracted in response to the increased resistance in arterioles. However, there was no significant difference in arteriolar diameter and RBC flow velocity between the BCCAO + EA + MLA group and the BCCAO + EA group, indicating that α-7-nAChR may not be involved in the regulation of arterioles by EA. The significant difference in venular RBC flow velocity between the BCCAO + EA + MLA group and the BCCAO + EA group may be attributed to blood stasis caused by inhibition of the anti-inflammatory pathway.

Nearly all ACh in the cortex originates from cholinergic neurons in the basal forebrain. The basal forebrain comprises multiple nuclei, including the medial septum, the horizontal and vertical limbs of the diagonal band of Broca (hDB and vDB), the substantia innominata, and the nucleus basalis of Meynert (Woolf, 1991). Among them, the hDB nucleus contains a large number of cholinergic neurons as its core component, which project extensively to the cerebral cortex (Chaves-Coira et al., 2016; Chaves-Coira et al., 2018) and are crucial for cognitive function (Wu et al., 2025). Therefore, neuronal activity in the hDB region of the basal forebrain indirectly reflects the level of ACh projection.

Therefore, cholinergic neuronal activity in the basal forebrain indirectly reflects the level of ACh projection. The percentage of c-Fos positive cells and the mean fluorescence intensity in the hDB nucleus region in basal forebrain of BCCAO + EA group mice were significantly higher than those in the BCCAO group, indicating that EA stimulation rescued the function of some neurons in the peripheral area of infarction, and may have further enhanced the functional compensation of healthy neurons. Previous studies have found that EA can increase the expression of c-Fos in the dorsal motor nucleus of the vagus nerve in rats with cerebral ischemia–reperfusion (Chi et al., 2018), while the observation of c-Fos expression in the basal forebrain is lacking. The ACh content in the basal forebrain of the mice in the BCCAO group decreased, while EA treatment significantly increased the ACh content. This indicated that EA stimulation resisted neuronal damage in the surrounding areas of infarction, thereby increasing ACh synthesis. This result is consistent with the changes of c-Fos protein in the basal forebrain.

Cholinergic neurons in the basal forebrain project extensively to the entire cortex. Specifically, the frontal cortex is associated with cognitive functions such as cognitive control, executive function (Friedman and Robbins, 2022), attention, working memory (Bahmani et al., 2019), abstract thinking, and decision-making ability (Chafee and Heilbronner, 2022). Therefore, we detected relevant indicators in the frontal cortex. The binding of ACh to acetylcholine receptors induces eNOS activation. eNOS mainly exists in vascular endothelial cells and is essential for vascular homeostasis and NO-mediated vasodilation (Farahani et al., 2025). When ischemic stroke occurs, the slow release of low-level NO mediated by eNOS can increase CBF and improve neurological function (Wu et al., 2023). Therefore, we selected eNOS as an observation indicator.

The results showed that EA stimulation significantly increased both the gene transcription levels and protein expression levels of m-1AChR, α-7-nAChR, and eNOS in the frontal cortex. In the BCCAO + EA + THP group, eNOS expression significantly decreased after the binding of ACh to m-1AChR was competitively inhibited. However, in the BCCAO + EA + MLA group, competitive inhibition of ACh binding to α-7-nAChR did not affect eNOS expression. These results indicate that m-1AChR plays an important role in the EA regulation of ACh/NO pathway in the frontal cortex of VaD mice, while α-7-nAChR was almost not involved in this process.

NO can dilate microvessels, increase cortical blood flow, and improve local metabolism, which is beneficial to the compensation and recovery of cortical function. EA stimulation significantly increased the NO content in the frontal cortex of VaD mice. Compared with the BCCAO + EA group, the BCCAO + EA + THP group had a significant reduction in NO content, while the BCCAO + EA + MLA group showed no significant change. This result was basically consistent with the changes in eNOS protein expression in the frontal cortex.

In recent years, researchers have elucidated mechanisms underlying acupuncture-induced improvement of CBF from multiple perspectives: modulation of astrocyte-related pathways (Ding et al., 2019), angiotensin II and its receptor-mediated signaling pathway (Li et al., 2014), maintenance of tight junctions between vascular endothelial cells (Wang et al., 2022), promotion of angiogenesis (Yang et al., 2025), and modulation of superoxide dismutase levels (Cui and Jia, 2022). These studies have contributed valuable explorations. However, few studies have elucidated the mechanism from the perspective of inter-regional brain connectivity and NVC.

Taking NVC as an entry point, we conducted a multilevel study—from macroscopic to microscopic scales, and from morphological to pathological analyses—to preliminarily explore the role of the basal forebrain cholinergic vasodilation system in EA-mediated regulation of cerebral microvessels in VaD mice. Nevertheless, our work has limitations. Due to constraints of time and funding, this study only assessed indicators after 7 days of EA intervention. In the future, we plan to further explore the long-term effect of EA, potentially conducting assessments at 2-week and 4-week timepoints post-intervention.

## 5 Conclusion

EA dilates cerebral arterioles by up-regulating the ACh/NO pathway within cholinergic vasodilation system, thereby alleviating cognitive impairment induced by VaD.

## Data Availability

The datasets presented in this study can be found in online repositories. The names of the repository/repositories and accession number(s) can be found in the article/supplementary material.

## Glossary

ACh: Acetylcholine
AD: Alzheimer’s disease
BCCAO: Bilateral common carotid artery occlusion
CBF: Cerebral blood flow
CCA: Common carotid artery
DI: Discrimination index
EA: Electroacupuncture
eNOS: Endothelial nitric oxide synthase
hDB: Horizontal limb of the diagonal band
m-1AChR: Muscarinic acetylcholine receptor 1
MLA: Methyllycaconitine citrate
NO: Nitric oxide
NR: Nitrate reductase
NVC: Neurovascular coupling
qRT-PCR: Quantitative real-time polymerase chain reaction
RBC: Red blood cell
THP: Trihexyphenidyl hydrochloride
VaD: Vascular dementia
WB: Western blot
α-7-nAChR: α-7-nicotinic acetylcholine receptor
